# The Sydenham Society of London

**Published:** 1856-12

**Authors:** Richard J. Dunglison

**Affiliations:** Hon. Local Secretary for Philadelphia


### *The Sydenham Society, of London.*

This Society was instituted in 1843, with the view of supplying its members with Standard Medical Works. The subscription, constituting a member, is five dollars annually, payable in advance. The following extracts from the Laws will explain the objects of the Society:

I. The Society is instituted for the purpose of meeting certain acknowledged deficiencies in existing means for diffusing medical literature, which are not likely to be supplied by the efforts of individuals, and shall be called the "SYDENHAM SOCIETY."

II. The Society will carry its objects into effect by a succession of publications, embracing, among others: 1. Reprints of standard English works, which are rare or expensive; 2. Miscellaneous selections from the ancient and from the earlier modern authors, reprinted or translated; 3. Digests of the works of old and voluminous authors, British and foreign, with occasional biographical and bibliographical notices; 4. Translations of the Greek and Latin medical authors, and of works in the Arabic and other Eastern tongues, accompanied, when it is thought desirable, by the original text; 5. Translations of recent foreign works of merit; 6. Original works of merit, which might prove valuable as books of reference, but which would not otherwise be published, from the slender chance of their meeting with a remunerating sale—such as bibliographies, alphabetical and digested indexes to voluminous periodical publications, &c.

Three volumes, handsomely bound in a uniform manner in cloth, gilt edged, are usually issued in the year.

List of the Society's Works, of which copies are still on hand, and from which new members, subscribing for the current year, may make a selection, on payment of an additional five dollars for any three volumes, with the exception of those to which an asterisk is affixed. Those to which an asterisk is affixed, or any other single volume, may be had for $2 50 per volume.

Sydenhami Opera Omnia, 1 vol.; Hasse's Pathological Anatomy, 1 vol.; Rhazes on the Smallpox and Measles, 1 vol.; The Works of Hewson, portrait and plates, 1 vol.; Dupuytren's Lectures on Diseases and Injuries of Bones, 1 vol.; Dupuytren on Lesions of the Vascular System, &c., 1 vol.; Memoirs of the French Academy of Surgery, 1 vol.; Feuchtersleben's Medical Psychology, 1 vol.; Microscopical Researches of Schwann and Schleiden, 1 vol., plates; the Works of W. Harvey, M. D., 1 vol.; the Genuine Works of Hippocrates, 2 vols.; Essays on Puerperal Fever, and other Diseases Peculiar to Women, 1 vol.; the Works of Sydenham, translated from the Latin, 2 vols.; Unzer and Prochaska on the Nervous System, 1 vol.; Annals of Influenza, 1 vol.; Romberg on Diseases of the Nervous System, 2 vols.; Kölliker's Manual of Human Histology, 2 vols., wood cuts; *Rokitansky's Pathological Anatomy, complete in 4 vols.; *Hunter on the Gravid Uterus, 1 vol. folio, 34 plates, with descriptive letter-press; Wedl's Pathological Histology, 1 vol., wood cuts; Oesterlen's Medical Logic, 1 vol.; Velpeau on Diseases of the Breast, 1 vol.; the Works of Aretæus, Greek and English, 1 vol.

RICHARD J. DUNGLISON, M. D.,
*Hon. Local Secretary for Philadelphia.*

